# Contemporary Aspects of Designing Marine Polysaccharide Microparticles as Drug Carriers for Biomedical Application

**DOI:** 10.3390/pharmaceutics15082126

**Published:** 2023-08-11

**Authors:** Paolina Lukova, Plamen Katsarov

**Affiliations:** 1 Department of Pharmacognosy and Pharmaceutical Chemistry, Faculty of Pharmacy, Medical University of Plovdiv, 4002 Plovdiv, Bulgaria; paolina.lukova@mu-plovdiv.bg; 2 Department of Pharmaceutical Sciences, Faculty of Pharmacy, Medical University of Plovdiv, 4002 Plovdiv, Bulgaria; 3 Research Institute at Medical University of Plovdiv, 4002 Plovdiv, Bulgaria

**Keywords:** polymer microparticles, marine polysaccharides, drug delivery, novel formulations

## Abstract

The main goal of modern pharmaceutical technology is to create new drug formulations that are safer and more effective. These formulations should allow targeted drug delivery, improved drug stability and bioavailability, fewer side effects, and reduced drug toxicity. One successful approach for achieving these objectives is using polymer microcarriers for drug delivery. They are effective for treating various diseases through different administration routes. When creating pharmaceutical systems, choosing the right drug carrier is crucial. Biomaterials have become increasingly popular over the past few decades due to their lack of toxicity, renewable sources, and affordability. Marine polysaccharides, in particular, have been widely used as substitutes for synthetic polymers in drug carrier applications. Their inherent properties, such as biodegradability and biocompatibility, make marine polysaccharide-based microcarriers a prospective platform for developing drug delivery systems. This review paper explores the principles of microparticle design using marine polysaccharides as drug carriers. By reviewing the current literature, the paper highlights the challenges of formulating polymer microparticles, and proposes various technological solutions. It also outlines future perspectives for developing marine polysaccharides as drug microcarriers.

## 1. Introduction

Traditional drug dosage forms and oral delivery formulations have limitations that have led pharmaceutical technology to focus on creating new drug-delivery systems. The main challenges facing contemporary pharmaceutical formulations include controlling the degree and rate of drug release to match the treated diseases, as well as directing the therapeutic agent to a specific site to enhance its effectiveness and minimize side effects. In recent years, using drug polymer carriers like microspheres, nanoparticles, or lipid structures has proven to be a successful approach in terms of improving drug delivery. Researchers worldwide are increasingly interested in the potential use of micro-sized drug carriers for targeted therapy. Many studies have shown the successful microencapsulation of various low molecular weight therapeutic agents, proteins, peptides, and nucleic acids. This proves that microparticles have the potential to be innovative drug-delivery systems, providing sustained/controlled drug release and targeted delivery in the body [1,2,3,4,5]. Microparticles are small structures, ranging from 1 to 1000 μm in diameter. Typically, they consist of a polymer matrix that incorporates the active substance. Depending on how the drug is distributed within the polymer, there are two general types of microparticles, as follows: microspheres and microcapsules. In the case of microspheres, the entire particle comprises a uniform mixture of the active substance and the polymer. On the other hand, microcapsules have a core drug substance that is coated with a polymer shell. The particle core can be solid, liquid, or even gas, and the active ingredient can form individual segments [6,7].

The characteristics of drug microcarriers, such as their size, shape, and encapsulation effectiveness, are determined by the materials and techniques used during the formulation process. These microstructures can be created using various monomeric or polymeric carriers and different production techniques, including spray drying, freeze-drying, coacervation, and emulsification methods. When constructing microparticles, the material used should have specific characteristics to ensure the active substance is effectively incorporated into the matrix. It should also provide high drug stability, control over drug release, and it should direct the drug to the desired site in the body. Many natural, semisynthetic, and synthetic polymers can be used as drug carriers for microparticle formulations. Polysaccharides from marine sources such as chitosan, alginate, carrageenan, and fucoidan are commonly used due to their biocompatibility, biodegradability, lack of toxicity, good encapsulating and mucoadhesive properties, and low cost [8]. Some marine polysaccharides also possess pharmacological effects such as antitumor, immunomodulatory, antioxidant, and anti-inflammatory activity, which can provide a synergistic therapeutic effect with the drugs used [9] in the microparticle.

The use of marine polysaccharides in drug delivery is highly beneficial due to their unique properties. They can be chemically or enzymatically modified to create various materials and they can be combined with proteins or other bioactive molecules to create drug-delivery systems that respond to stimuli. Marine polysaccharides can also form interpenetrated polymeric networks, which can effectively control the release rate of the drugs they contain [10]. This allows for lower dosages of drugs to be used, thus reducing the risk of side effects. Additionally, marine polysaccharides are useful for gene therapy due to their ability to stabilize and protect genetic material and other therapeutic agents, as well as improve drug solubility and promote sustained release [11].

In recent years, literature reviews have summarized the use of marine polysaccharides as drug carriers. However, these reviews mainly focus on the formulation of drug-loaded nanostructures, and they lack detailed data on microstructures [12,13,14]. Nano-sized carriers have their advantages, but in some cases, microparticles are preferred. The size difference between micro- and nanoparticles has various effects. Smaller particles have more free surface areas and are more likely to aggregate, which can affect their stability. Smaller particles are also more accessible for the drug, with regard to the external aqueous phase during production, resulting in lower drug loading for smaller particles. Additionally, water can penetrate smaller particles more quickly, leading to an increased drug burst release and faster release kinetics [15]. Furthermore, the rapid penetration of water in the polymer can cause the faster deterioration of particles, which can also hasten the release of drugs. Microparticles can improve the control and delay of drug release and enhance local drug delivery. Generally, microparticles are less likely to penetrate most biological barriers compared with nanoparticles. Therefore, they often need to be directly delivered to the target site, where they can form a depot and mainly provide local and extended effects [15]. Microparticles are also preferred in certain administration methods over nanoparticles. For instance, nasal dry powder formulations require micro sized particles for deposition in the nasal cavity [16].

This review paper aims to provide an overview of the current progress in designing microparticles that use marine polysaccharides as drug carriers. To the best of our knowledge, there have been no reviews on this topic in the literature so far. By investigating the contemporary literature, we have highlighted the essential challenges in formulating polymer microparticles, we have proposed various technological solutions, and analyzed future perspectives for developing polysaccharide drug microcarriers.

## 2. Polysaccharide Drug Carriers

### 2.1. Polysaccharide Classification, Sources, and Isolation

**Figure 1:**
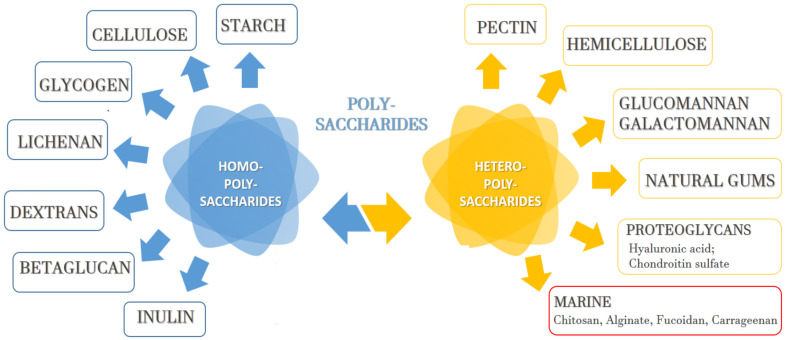
Classification of natural polysaccharides. Created by biorender.com (publication license accessed on 22 June 2023).

Polysaccharides can be classified in accordance with their structure, chemical composition, and sources. They are defined as polyhydroxyketones or polyhydroxyaldehydes, which are composed of three to seven carbon atoms. The polymer chains consist of monosaccharide residues connected through O-glycosidic bonds [17,18]. Based on their composition, there are homopolysaccharides/homoglycans—these contain only one type of monosaccharide unit. However, heteropolysaccharides/heteroglycans can also consist of different monosaccharides (Figure 1). Natural polysaccharides can be obtained from plants, algae, lichens, fungi, animals, and some microorganisms by using different methods for extraction, purification, and separation [19]. The most commonly applied polysaccharide extraction techniques include extraction with hot water, extraction with alkalized water, extraction with a dilute mineral acid, and extraction using enzymes. To isolate the polymers located in the cell wall (intracellular or endopolysaccharides), fragmentation of the raw material is necessary, which can be performed mechanically, using ultrasounds, or gas flow [20,21]. Crude polysaccharide extracts usually contain impurities such as inorganic salts, lipids, proteins, and low-molecular non-polar substances. A dialysis process is usually used to remove low molecular weight impurities. Proteins can be separated via a protease method, called the Sevag method, using trifluoroacetic acid or trifluorotrichloroethane. Lipids can be removed with organic solvents such as ethanol, ether, or petroleum, whereas the pigment purification is usually performed via adsorption and oxidation processes [19]. Extracts, composed of different polysaccharide molecules, can be obtained in the extraction process. Therefore, various precipitation methods, ultracentrifugation, electrophoresis, chromatographic methods, as well as other biochemical methods, are applied for their separation [22].

### 2.2. Polysaccharide Molecular Weight, Microparticle Size, and Drug Release

The resultant polysaccharides as a final product are characterized by their molecular weight, monosaccharide composition, and total sugar content [17,18,19]. Polysaccharides usually have a high molecular weight and can establish multiple inter- and intra-molecular interactions due to their free hydroxyl groups. Thus, they can significantly increase the viscosity of the medium and cause its gelling. These characteristics of the polysaccharides are essential for their particle-forming ability and for the mechanism through which the polymer matrix/shell swells/degrades and releases the incorporated active substance. Polymers can be divided into two general types depending on their functional groups and how they interact with water. Some of the polysaccharides allow water to penetrate them, resulting in the degradation of the entire microparticle matrix. In this case, an initial burst release of the incorporated drug substance is observed, followed by a sustained diffusion-controlled release [23,24]. The other group of polymers consists of surface-degrading molecules. They are mainly composed of hydrophobic monomers connected by weak bonds. These polymers do not allow water to enter the core of the particles, and they degrade gradually via hydrolysis into oligo- and monomeric structures, but only on the surface between the polymer and the water [25,26]. Regardless of the molecular weight of the polysaccharide used, all microparticles initially released via rapid diffusion were low molecular oligomers. Regarding particles composed of low-molecular weight polymers (for example, polysaccharides with a molecular weight below 50 kDa), the number of decaying products usually released from the matrix increases over time, whereas for microparticles composed of high-molecular polymers (polysaccharides with molecular weights higher than 300 kDa), it could be constant for a longer period of time. Polysaccharide carriers with higher molecular weights are associated with a slower diffusion rate, and hence, a slower drug release rate. Low molecular weight polymers form pores during the process of degradation more quickly; through these pores, the active substance can easily diffuse out of the carrier [25,26,27]. 

The molecular weight of the polysaccharides that were used as drug carriers also affected the size and porosity of the resultant microparticles. Polymers with a larger molecular weight formulate more viscous stock solutions in smaller concentrations, which usually produce particles of larger sizes. Moreover, the microparticles’ size strongly affects the drug release rate. As the microparticle size decreases, the ratio between the particles’ surface area and volume increases. Thus, at a certain diffusion rate, the smaller size of the microcarriers leads to the faster passage of the drug substance through a certain mass of the dosage form. Moreover, water enters smaller particles easily due to the smaller distance between their surface and their center. The reduced surface area of particles with larger sizes may lead to the slower degradation of some less water-permeable polymers [28]. The porosity of the formulated microparticles can be controlled by the method for their preparation, and drug substances are released significantly faster from a highly porous polymer matrix compared with non-porous ones [29].

### 2.3. Biodegradability and Mucoadhesiveness of Polysaccharide Microcarriers

Two of the most essential characteristics of drug-delivery systems are biodegradability and biocompatibility. Biodegradability is of utmost importance for the prevention of acute or long-term toxicity. Natural polysaccharides have been proven as biodegradable polymers, and under physiological conditions, their chains can be easily broken by various enzymes in the mucous surfaces, in the stomach or produced by the normal intestinal flora [30,31]. Studies have shown that the degradation of natural polysaccharides does not lead to the accumulation or retention of decaying substances in the body. The products of their metabolism are oligosaccharides, which either enter the metabolic pathways of glycosaminoglycans and glycoproteins, or they are directly excreted through the kidneys [8,32]. The mucoadhesive properties of polysaccharides are essential for developing polymer dosage formulations for buccal, nasal, vaginal, and rectal administration. The use of mucoadhesive polymers allows for the localization of the drug substance at the absorption site, an extended contact time between the formulation and the targeted biological tissue, as well as improved drug bioavailability [33]. The development of adhesive systems depends on the properties of the polymers used. Most natural polysaccharides like chitosan, alginate, fucoidan, and so on, possess excellent mucoadhesive properties. They interact with the mucous surface through non-covalent chemical bonds—hydrogen, van der Waals, or ionic bonds—ensuring the polymer’s effective attachment to the targeted tissue [34]. Therefore, polysaccharides are becoming promising drug microcarriers that can successfully direct active substances to the mucous tissues in the body, such as gastrointestinal, oropharyngeal, ophthalmic, buccal, and nasal surfaces.

### 2.4. Marine Polysaccharides for Microparticle Formulation

**Figure 2:**
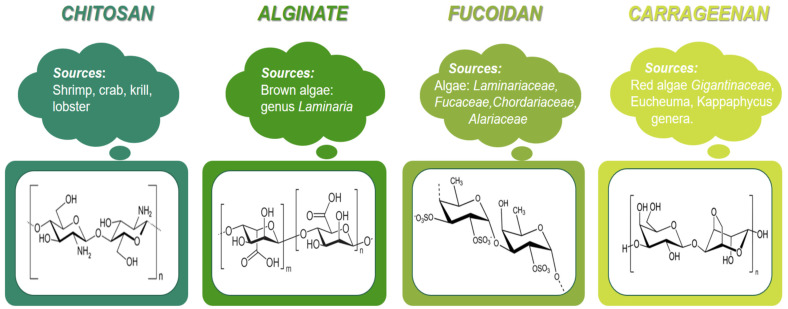
Chemical structure and sources of the marine polysaccharides chitosan, alginate, fucoidan, and carrageenan. Created by biorender.com (publication license accessed on 22 June 2023).

Generally, to be a promising candidate for a drug microcarrier, the polymer should be biocompatible, biodegradable, non-toxic, able to provide modified and targeted drug release, and it should offer increased drug stability. Most marine polysaccharides meet these requirements. They are affordable and allow relatively easy production of microsystems with high drug entrapment efficiency. Such polysaccharides are chitosan, alginate, fucoidan, carrageenan, and so on, which have been intensively studied as drug carriers in recent years (Figure 2).

#### 2.4.1. Chitosan Microparticles

Chitosan can be produced via the chemical deacetylation of chitin derived from crustaceans [35]. The most common sources for its industrial production are shrimp, crab, lobster, and krill. Historically, chitin was first isolated from fungi, and later from beetle cuticles. Recently, there has been increased interest in chitosan derived from fungi (*Mucor rouxii*, *Aspergillus niger*, *Penicillum crysogenum*, *Lactarius vellereus*) and insects (ladybug, silkworm, butterflies) [18]. Although highly affordable, chitin has limited uses, mainly due to its insolubility in water. For that reason, it is primarily processed into chitosan, the structure of which is soluble in an aqueous medium with an acidic pH. Chitin is a polymer composed of N-acetyl-D-glucosamine. When its molecules are deacetylated and the repeating units in its composition are mostly free of acetyl functional groups such as β-1,4-D-glucosamine, the polymer is known as chitosan [35,36,37]. The deacetylation process is carried out to different extents, depending on the desired application, to obtain products with varying degrees of deacetylation (DD).

Different chitosan derivatives can be obtained via chemical modifications of the polysaccharide reactive units. The polymer can be functionalized via different mechanisms using its free amino and hydroxyl groups, which can participate in various chemical reactions [36]. Some of the most common modification techniques include phosphorylation, thiolation, N-phthaloylation, and crosslinking [37]. Chemical modifications can improve the physical and chemical properties of chitosan and extend its possible applications. Chitosan has been outlined as one of the most mucoadhesive polymers, and it is also characterized by its permeability-enhancing properties, which is related to its ability to facilitate the paracellular transport of hydrophilic molecules by opening the dense bonds in the mucosal barriers [38]. Furthermore, studies have reported its antimicrobial [39] and antioxidant [40,41] activity. 

Chitosan microparticles can be obtained through the widely-used method of spray drying. He et al. utilized this method to develop chitosan microparticles as drug-delivery systems for cimetidine, famotidine, and nizatidine. The results revealed that the particle size increased when a nozzle with a broader diameter was used. Conversely, increasing the gas flow rate produced smaller microspheres. Additionally, inlet air temperature between 140 °C and 180 °C did not significantly affect particle size. [42]. The spray drying method for preparing microparticles with chitosan often results in low yields due to the adhesive properties of the polysaccharide. In a study by Cevher et al., chitosan microspheres with vancomycin hydrochloride were produced using a Mini Spray Dryer B-191, Büchi, from a 1% (*v*/*v*) acetic acid solution containing different polymer:drug ratios (1:1, 2:1, 3:1, and 4:1 *w*/*w*). The yield obtained was between 47% and 50%, which was attributed to the small sample solutions used for spray drying (200 mL) [43]. It is also possible to obtain chitosan microparticles using emulsion techniques. In accordance with a study conducted by Pilicheva et al., betahistine-loaded chitosan microspheres were formulated using the W/O emulsion solvent evaporation technique. These microparticles had a high drug loading and entrapment efficiency, and they were able to release the incorporated drug in a sustained manner [44]. Complex coacervation is another method for producing microspheres from chitosan via ionic interactions between the polysaccharide and another oppositely charged polymer. When creating coacervates with chitosan, the most frequently utilized polymers are sodium alginate, sodium carboxymethyl cellulose, and the sodium salt of polyacrylic acid. Bayomi et al., for example, developed diltiazem-loaded microspheres using chitosan and casein using the colloid coacervation method [45]. The formation of particles occurred due to an interaction between the solution of chitosan in acetic acid and a casein solution in a sodium base. Formaldehyde was used as a crosslinking agent. The concentration of both polymers and the drug substance, as well as the stirring rate, were found to affect the properties of the particles. The microparticles obtained using this method had a broad size distribution and they tended to aggregate.

Chitosan is a widely used polymer for the development of drug-loaded microsystems for oral [46,47,48,49], dermal [50,51,52,53,54], nasal [55,56,57,58,59,60], and ophthalmic [61,62,63] administration (Table 1). Due to their good adhesion to the oral mucosa and gingiva, chitosan microparticles have been developed for potential application against periodontitis, oral candidosis, cavities, and other dental conditions [46,47,48,49]. Furthermore, polysaccharides exhibit activity against dental plaque, which makes it applicable in dental practice settings [64]. Chitosan can destroy bacterial cells by enhancing the displacement of Ca^2+^ from anionic sites along the cell membrane [18]. The polysaccharide has been reported to be effective against certain bacteria in the oral cavity such as *Porphyronomas gingivalis*, *Prevotella intermedia*, and *Actinobacillus actinomycetemcomitans* [65,66]. Chitosan shows an inhibitory effect against *S. salivarius* and *S. sobrinus*, as it can reduce up to 93.4% of the biofilm formation of *S. mutans*, which plays a vital role in the pathogenesis of dental cavities [67].

Chitosan microparticles can improve the dermal absorption of low molecular weight polar drug substances, peptides, and proteins [50]. They are also used as formulations for wound healing and skin regeneration. An additional advantage is the hemostatic and anti-inflammatory activity of the carrier [68]. Such structures can also allow transfollicular drug delivery, overcoming the skin barrier by passing through hair follicles [48]. Chitosan microparticles have been used in cosmetics and therapeutic products to work against acne [69], and as carriers of sunscreen agents with hydrophilic properties [54]. 

Many examples in the literature demonstrate the use of chitosan as a carrier of drug substances for ophthalmic applications. The positive charge of chitosan enables the polymer to interact with the negatively charged cornea and the eye’s conjunctiva, thus achieving the longer retention of the ophthalmic dosage form on these eye structures and providing a higher local drug concentration. Moreover, chitosan can increase drug absorption, even of polar molecules with higher molecular weights, by temporarily expanding the spaces between the corneal epithelial cells. This marine polysaccharide has antibacterial and healing properties, it does not cause toxicity, and does not irritate the eye when applied topically [70,71]. The following ophthalmic chitosan microparticles have been included: tetracycline [61], atropine [62], acyclovir [63,72], among others.

Illum et al. were among the first scientists to demonstrate that chitosan can significantly increase absorption across the nasal epithelial membrane of both small polar molecules and larger peptides and proteins [73]. Their studies led to increased interest in nasal chitosan drug formulations like chitosan solutions, powders, gels, nano-, and microparticles. Applied in the nasal cavity, chitosan microparticles form a gel on the nasal mucosa, and depending on the preparation method and their characteristics, they can provide a sustained release of the drug they carry. Chitosan particles adhere for a longer time after administration compared with a nasal solution of the polymer. If their size is above 10 μm, there is no risk of reaching the lungs after application [74]. Chitosan microparticles can be obtained via various preparation methods, such as spray drying or emulsion techniques, which are usually followed by the crosslinking of the polymer carrier. They can be applied in the form of a dry powder to the nasal cavity, and they have the ability to absorb water and form a mucoadhesive gel. Thiolation of the polymer carrier is a successful approach for enhancing the mucoadhesive properties of chitosan microparticles and improving transmucosal drug delivery [75]. The most common method for synthesizing thiolated chitosan involves covalently conjugating ligands loaded with thiol groups to the polymer base through the formation of amide structures. The presence of thiol groups dramatically increases the mucoadhesive properties of the polymer, as it forms strong covalent disulfide bonds with cysteine-rich parts of the mucous layer [76,77]. Thiolation also significantly affects other properties of the polymer, such as cohesion and permeability. Free thiol groups can form disulfide bonds between polymer chains and between separate sections of one polymer chain. This cross-linking process gives the thiomers significant cohesive properties, and it further reinforces their level of binding to the mucus [78]. In addition to having more pronounced mucoadhesive properties, chitosan thiomeres exhibit a greater enzyme-inhibiting activity, blocking metal ions from the enzyme structures [79]. Regarding chitosan microparticles for nasal administration, they have been successfully included in various low-molecular drug substances such as methotrexate [55], ketorolac [56], isosorbide [59], gentamicin [80,81], betahistine [82], metoclopramide [83], rivastigmine [84], verapamil [85], deferoxamine [86], antimigraine drugs (ondasetron) [57], zolmitriptan [58], as well as proteins such as insulin [60,87]. 

**Table 1:** Chitosan-based microparticles as drug delivery systems.

Active Substance	Administration	Reported Results	Reference
Ornidazole	Oral	Mean diameter, 29.1–52.65 µm; Drug encapsulation, 11–32%; Sustained drug release up to 5 days; Inhibition of the growth of *Staphylococcus aureus*.	[42]
Metronidazole	Oral	Free-flowing spherical particles with an average size of 42.82 μm; Drug entrapment efficiency of 59.40%; Prolonged in vitro drug release profile.	[43]
Metronidazole	Oral	Spherical, rough, and porous particles; Average size of 800 µm; Drug entrapment efficiency of 60–75%; Percentage swelling, 10–25%; Bioadhesion, 43–59%.	[44]
Ketoprofen	Oral	Microparticles with narrow size distributions; Mean diameter, 2.11–3.27 µm; Good sphericity and a smooth surface; Linear in vitro drug dissolution behavior.	[45]
Ascorbic acid Nicotinamide	Dermal	Sustained drug release profile; Ex vivo skin retention of the drugs in the epidermis/dermis; Time- and dose-dependent antibacterial activities.	[46]
Ampicillin	Dermal	Spray-dried microparticles with an encapsulation efficiency of 85%. Good wound healing properties, leading to rapid cicatrization.	[47]
Minoxidil sulfate	Dermal	Encapsulation efficiency of 82%; Mean diameter of 3 µm; Spherical morphology without porosities. Intensive swelling and sustained drug release.	[48]
Catechins	Dermal	Chitosan microparticles significantly improve the ability of catechins to permeate the skin and effectively prevent their enzymatic degradation.	[53]
Phenylbenzimidazole sulphonic acid	Dermal	Production yield, 76%; Average size in the range of 24–100 μm; Entrapment efficiency, 29–74%; Sustained release over 8 h in accordance with a biphasic pattern; Improved in vitro UV screening effect.	[54]
Methotrexate	Nasal	Entrapment efficiency of 90–99%; Average size, 3.3–4.9 μm; Prolonged drug release; Nasal ciliotoxity shows only minor cilia irritation.	[55]
Ketorolac	Nasal	Drug encapsulation efficiency, 52–78%; Particle size, 14–46 μm; Prolonged drug release, fitted in accordance with the Higuchi model using Fickian diffusion; No severe damage to the integrity of nasal mucosa after ex vivo experiments.	[56]
Ondasetron	Nasal	Sustained drug release for 24 h; In accordance with in vivo data on rats, the particles attain a sustained plasma profile with significantly larger area under the curve.	[57]
Zolmitriptan	Nasal	Spray-dried spherical microparticles with a narrow size distribution; Production yield of 40–76%; Entrapment efficacy of 93–105%.	[58]
Isosorbide dinitrate	Nasal	Improved intranasal drug absorption in accordance with in vivo studies on rats; Good safety profiles according to the results of nasal ciliotoxicity tests.	[59]
Insulin	Nasal	Controlled drug release over 6 h; Absolute bioavailability of 7.24% after nasal administration on conscious rats.	[60]
Gentamicin sulfate	Nasal	Mean particle size of 29.47 μm; Drug loading of 13.32%; Good mucoadhesive properties evaluated by determining the mucociliary transport rate across a frog palate.	[80,81]
Betahistine dihydrochloride	Nasal	Microspheres with a spherical shape, smooth surface, a mean size of 3.82–7.69 μm; Sustained drug release; Good mucoadhesive properties.	[82]
Metoclopramide	Nasal	A mean particle size of 3–10 μm; Good in vitro mucoadhesive properties; In vitro release profiles within the range of 1–3 h; High ex vivo drug permeation through the nasal mucosa.	[83]
Rivastigmine	Nasal	Particle size of 19.9 µm; Entrapment efficiency of 77.8%; Drug release, T_80%_ of 7.3 h; In vivo enhanced nose-to-brain delivery in rats.	[84]
Verapamil hydrochloride	Nasal	Spherical microparticles with sizes in the range of 21–53 μm; High drug entrapment efficiency; Burst followed by sustained release over 6 h; Bioavailability, 58.6%.	[85]
Insulin	Nasal	Mean particle size, 20–45 µm; Insulin loading, 4.7–6.4%; Sustained drug released following a Higuchi model.	[87]
Tetracaine hydrochloride	Ophthalmic	Minimum cytotoxicity; Optimum cellular uptake; Significantly increased duration of drug action (up to a fourfold increase).	[61]
Atropine sulfate	Ophthalmic	Ideal physicochemical characteristics for ophthalmic application; Superior in vivo effects of the microparticles on mydriasis in rabbits compared with solutions.	[62]
Acyclovir	Ophthalmic	Encapsulation efficiency, 75%; Only mild tissue damage, in accordance with the results of an irritation (SMI) assay.	[63]
Acyclovir	Ophthalmic	Drug loading efficiency, 76.99–97.86%; In vitro sustained release for 12 h; No signs and symptoms of ocular toxicity in accordance with a tolerance study in rabbit eyes.	[72]

#### 2.4.2. Alginate Microparticles

Alginates are widely used natural polymers in pharmaceuticals, usually applied as viscosity-enhancing, gel-forming, and stabilizing agents. Alginate matrix or membrane microstructures are commonly used as drug delivery systems. Alginate microparticles are biocompatible, biodegradable, and non-toxic drug carriers that are applied to encapsulate hydrophilic and hydrophobic active substances, including bioproducts and cells. Alginates are natural water-soluble polysaccharides extracted from the cell walls of various types of brown algae. They are linear copolymers of D-mannuronic acid (M block) and L-guluronic acid (G block) bound by β-1.4 glycosidic bonds. These monomers can be arranged in homogeneous (poly M, poly G) or heterogeneous (poly MG) configurations. The structure of alginates depends on their origin, the type of algae (mainly *Macrocystis pyrifera*, *Laminaria digitata* and *Laminaria saccharina*), geographical location, as well as seasonal and annual features [88].

Univalent metal ions can form soluble salts with the alginate, whereas divalent and multivalent cations, like Ca^2+^, crosslink the polymer chains in gel systems. Crosslinking allows the formulation of alginate particles with different sizes. This process is relatively easy, and is performed by adding a solution containing calcium ions to a sodium alginate solution and replacing Na^+^ with Ca^2+^. Each Na^+^ cation binds to only one carboxyl group of the alginate chain, whereas Ca^2+^ interacts with two such groups from different polymer chains. The polymerization reaction is due to the crosslinking of copolymers using ionic bonds between calcium cations and alginate anions. The specific structure resulting from this interaction is called the “egg-box” model [2].

As mentioned above, the viscosity of the polymer solution is an essential parameter in the microencapsulation of drug substances. It affects the physicomechanical characteristics of the resulting microparticles and the efficiency of drug incorporation into the microparticles. The viscosity of alginate solutions depends on various factors such as the pH of the medium, the molecular weight, and the concentration of the polymer [5]. With a decrease in pH, the carboxyl groups in the structure of the polysaccharide are protonated and they form hydrogen bonds. This leads to an increase in the viscosity of the solution. It reaches maximum values around pH 3–3.5. The average molecular weight of sodium alginate is 216.121 g/mol. With an increase in the molecular weight of the polymer, the gelling rate increases, as does the strength and elasticity of the formed hydrogel [89,90]. 

Alginate possesses good mucoadhesive properties due to the presence of free carboxylic and hydroxyl groups in its structure. In a physiological environment, electrostatic repulsion forces occur between alginate and mucin due to the negative charges of sialic acid and sulfate groups in the structure of mucin and the alginate anionic carboxyl groups. Therefore, adhesion is achieved, not via electrostatic interactions, but intermolecular hydrogen bonds [91]. The good mucoadhesive properties make alginate microparticles suitable for topical application to the skin and biological mucous membranes, such as nasal and buccal membranes [92]. 

Studies reported that alginate microspheres can improve the oral delivery of different groups of therapeutic agents. Such polymeric structures, for example, have been used as carriers for ranitidine [93], acyclovir [94], isoniazid [95,96], metformin [97], caffeine [98], insulin [99], as well as various nonsteroidal anti-inflammatory drugs, as follows: diclofenac [100], indomethacin [101], aceclofenac [102], and piroxicam [103] (Table 2). In vivo tests have shown that alginate microparticles can be retained on the gastric mucosa for more than 4 h, providing a longer residence time in the stomach [94]. Gamma scintigraphic studies were conducted to determine the alginate particles’ location after oral administration and the gastrointestinal passage rate. After oral administration, alginate microspheres were observed in the intestines, even after 24 h, which can define these structures as suitable systems for targeted drug delivery in the gastrointestinal tract [95]. 

Bioadhesive sodium alginate microspheres have also been successfully administered for intranasal systemic drug delivery [104,105]. Studies on animals have shown that such systems can significantly improve therapeutic efficacy and provide better control over the treated symptoms [104]. In vitro adhesion tests on nasal mucosa indicated that even when crosslinked with varying amounts of CaCl_2_, alginate microparticles had satisfactory mucoadhesive properties and could be successfully administered nasally. On the other hand, higher CaCl_2_ concentrations and longer crosslinking times negatively affected the adhesion of the microspheres. When crosslinked, the mobility of the polymer chains decreases. Crosslinked microspheres absorb water, but they are insoluble and do not form a hydrogel layer on the nasal epithelium. They retain a more rigid gel structure upon contact with the mucous membrane [105].

Alginates are also used as a microencapsulation material for probiotic bacterial strains. They can form an extremely flexible, biocompatible, biodegradable, and non-toxic coating, protecting the active components from external factors such as heat and moisture, thereby enhancing their stability and bioavailability [106,107]. On the other hand, the low mechanical strength of alginate particles, and the large size of their pores, can cause the leakage of biomolecules from the microcapsules. This disadvantage can be overcome, and the encapsulation efficiency can be increased by incorporating capsule wall sealing agents into the capsule (e.g., chitosan, peptides, or fructooligosaccharides) [108]. Through alginate microcapsules, the effective delivery of probiotic microorganisms can be achieved. Alginates increase the survival of probiotic bacteria both in the product and in the gastrointestinal tract, allowing them to reach the intestines, and thus, enabling them to exert a positive effect on the microbiome [109]. Alginates exhibit a prebiotic effect on low molecular weight sugars, and they may extend the shelf life of probiotic products [110].

#### 2.4.3. Fucoidan Microparticles

Fucoidans are sulfated polysaccharides that mainly consist of fucose repeating units (90% of the total sugar composition) and other sugar monomers, such as galactose, mannose, glucose, and uronic acids [111]. The presence of sulfate ester groups provides a negative charge on the macromolecule structure that is responsible for the anionic characteristics of the polymer. Fucoidans are derived from brown seaweed. They act as structural polysaccharides in the cell walls of brown macroalgae, but they have also been found in echinoderms and some lower plants [112]. 

Due to its specific structure, composition, and various biological effects (antioxidant, antibacterial, antiviral, anticoagulant, hypolipidemic, hypoglycemic, antitumor, anti-inflammatory and immunomodulatory activities), fucoidan has been outlined as a promising therapeutic agent and an excipient for various pharmaceutical formulations, including microparticles (Table 3) [113,114,115,116].

Microsized drug-delivery systems that are developed using fucoidan refer to the term fucospheres. Fucospheres can be formulated via various physical, physicochemical, or chemical methods. Usually, the production process involves the addition of copolymers—for example, crosslinking fucoidan with positively charged chitosan [117,118]. Results have indicated that the size of the prepared microparticles can be increased by using higher polymer concentrations [119]. The increase in fucoidan content can also lead to the greater zeta potential of the formulated particles and a higher drug encapsulation efficiency [118]. 

Fucoidan microparticles have been formulated as oral and vaginal delivery systems for the sustained release of antibiotics and antifungal agents. For example, ofloxacin [118] and posaconazole [120] were incorporated into fucospheres using different preparation methods—spray-drying, complexation, and precipitation techniques. Both formulations exhibited good physicomechanical characteristics, high entrapment efficiency, and prolonged drug release.

Fucoidan-based microparticles have been considered potential pulmonary drug delivery systems for the treatment of tuberculosis. Fucoidans can be suitable carriers for pulmonary drug delivery due to the ability of the polysaccharide to recognize macrophages in the alveoli [121]. The antituberculosis agents, isoniazid and rifabutin, were successfully included in fucoidan microparticles for pulmonary administration [122]. The formulated systems were characterized, and were relatively safe, as per MTT tests, for the human alveolar epithelium; they exhibited high efficacy against *Mycobacterium tuberculosis*.

Fucospheres have also been investigated for dermal applications when treating dermal burns [119]. In vivo studies demonstrated that such microstructures can provoke strong wound-healing effects and rapid epithelization. The increased skin regeneration induced by the fucospheres was likely due to the effect of fucoidan on the fibroblast migration, the release of growth hormones, and the cytokines involved in the re-epithelization process. 

Fucoidan can be used as a coating polymer for microparticles, providing mucoadhesive properties. MTT cytotoxic assays showed that fucoidan-coated microcapsules did not affect cell viability [123]. Moreover, this polysaccharide can bind to P-selectin, which is a key protein involved in the activation of platelets associated with cancer cell metastasis. Therefore, the inhibition of P-selectin is an essential mechanism for establishing an effective anticancer therapy [124]. The ability of fucoidan to target P-selectin proteins is another reason to use this polysaccharide as a polymer carrier in oncology. In vivo animal tests have also demonstrated its polymer adhesiveness and affinity towards the protein. Moreover, outlined fucoidan microstructures can be used as targeting systems, which can be used in therapy and diagnostics [123]. Fucoidan-coated microparticles, loaded with doxorubicin, are another example of the potential of such formulations, with regard to drug-delivery systems for cancer therapy [125].

**Table 3:** Fucoidan-based microparticles as drug delivery systems.

Active Substance	Administration	Reported Results	Reference
Ofloxacin	Oral	Average particle size, 0.61–1.48 µm; Zeta potentials, 5.6–28.0 mV; Release mechanism fitted in accordance with the Higuchi kinetic model.	[118]
Posaconazole	Vaginal	Good mucoadhesive properties; High drug loading; Sustained drug release in simulated vaginal fluid (after 8 h, 65.34%; in pH, 4.2; and 33.81% in pH 1.2).	[120]
Isoniazid, Rifabutin	Pulmonary	Median particle diameter, 3.6–3.9 µm; Entrapment efficiency of isoniazid (97%) and rifabutin (95%); No cytotoxic effects on lung epithelial cells.	[122]
Fucoidan	Dermal	Microparticle size of 1017 µm; Bioadhesion, 0.081–0.191 mJcm^−2^; Surface charges, +6.1 to +26.3 mV; Improved skin regeneration and re-epithelization.	[119]
Perfluorooctyl-bromide	Parenteral	Core–shell structures with sizes, 2–6 µm; Stable in storage over 30 d at 4 °C; High specific binding efficiency to P-selectin and activation of platelet aggregates.	[123]
Doxorubicin	Parenteral	Particle size, 1.91–2.03 μm; Drug encapsulation efficiency of 69.7%; Drug-controlled release; Significant antiproliferative efficiency in breast cancer cell lines.	[125]
Bovine serum albumin	Parenteral	Smooth and spherical microspheres with sizes in the range of 0.61–1.28 µm. Drug encapsulation efficiency, 51.8–89.5%; In vitro three-phasic sustained drug release pattern.	[126]

#### 2.4.4. Other Marine Polysaccharides as Potential Microcarriers

Carrageenans are sulfated polysaccharides, which are members of the red algae *Gigantinaceae* family, and the *Eucheuma* and *Kappaphycus* genera. They are composed of galactose residues, connected by alternating glycosidic bonds, with different degrees of sulfatation. Due to the negative charges of the sulfated groups, carrageenans are classified as polyanions. They exhibit a protective activity against fungi, viruses, and bacteria [127]. Carrageenans have been used for the oral drug delivery of cell therapies, as well as for cell encapsulation and cartilage regeneration applications (Table 4) [128,129,130,131,132,133,134,135,136]. Carrageenans are considered safe, and they are allowed for oral administration, both as pharmaceutical carriers or as food additives by the Food and Drug Administration (2018), the European Parliament and Council Regulation (No 1333/2008, Annex II and Annex III), and the Joint FAO/WHO Expert Committee on Food Additives (JECFA) [137]. However, therapeutic applications of low molecular weight carrageenans have been limited because of their possible gastrointestinal toxic side effects [138]. Furthermore, carrageenans can cause inflammatory responses when injected, including localized edema, infiltration of white blood cells, increased levels of local PGE2, and increases in interleukin-8 (IL-8) secretion [139].

Ulvans are another group of sulfated marine polysaccharides, isolated from green algae, from the *Enteromorpha* and *Ulva* genera. Their polymer chain contains xylose, glucose, iduronic acid, glucuronic acid, and rhamnose residues, and they can have different charge distributions, densities, and molecular weights [140]. Ulvans exhibit immunomodulatory, antitumor, antiviral, antioxidant, antihyperlipidemic, and anticoagulant biological effects, and they have been used as chelating agents in wound healing treatments and in the development of various drug-delivery systems [141,142].

Chondroitin is a polysaccharide, composed of N-acetyl galactosamine and glucuronic acid units. Although it has been mostly extracted from non-marine sources, it can also be isolated from sharks, whales, salmon fish, some cnidarians, mollusks, and sea cucumbers. The polysaccharide possesses strong anticoagulant properties, and it has also been administered as a supplement for preventing arthritis. Chondroitin can be used in the formulation of hydrogels for the regeneration of cartilage tissue [143,144,145].

Hyaluronan is a polyanionic heteropolysaccharide, which can be isolated from various marine animal sources, like vitreous humor and cartilages of different fish species. Its structure mainly consists of repeating N-acetyl-D-glucosamine and D-glucuronic acid disaccharide units [146]. Hyaluronans have been used for a wide variety of biomedical applications. They are a biological marker for rheumatoid arthritis and can be administered as supplements for arthritic patients [147]. These polysaccharides affect cell proliferation, differentiation, and migration, and therefore, they can be utilized for wound healing and tissue regeneration [148]. Due to their negative charge, hyaluronans can be used for formulating microspheres via complexing with cationic polymers [149]. Hyaluronic acid microparticles, and cross-linked hyaluronan-chitosan microspheres, have been reported as a diagnostic tool and as reservoir systems for several bioactive agents [150,151,152,153].

Although not thoroughly investigated in the pharmaceutical field due to difficulties with their extraction, some emerging glycosaminoglycan-like polysaccharides with marine origins may show promising potential for drug carriers when developing micro- or nano-sized drug delivery systems. Such polysaccharides are dermatan, keratin, agarose, heparin sulfate, and so on.

**Table 4:** Microparticles, based on marine polysaccharides, formulated as drug delivery systems.

Drug	Polysaccharide	Reported Results	Reference
Ibuprofen	Carrageenan	Average particle size of 15.97 μm; Drug loading, 35–70%; Pore size, 8.5–13.5 nm; Amorphous form of the incorporated drug with an enhanced in vitro release profile.	[129]
CoQ10.	Carrageenan	Incorporation of CoQ10 into carrageenan microcapsules resulted in amorphous powder with significantly higher water solubility compared with pure CoQ10.	[130]
Insulin	Carrageenan	Drug encapsulation efficiency of 94.2%; Drug loading capacity of 13.5%; Prolonged hypoglycemic effect, up to 12–24 h, after oral administration in diabetic rats.	[131]
VEGF, Eumenitin	Carrageenan	Particle diameter, 295 μm; Sustained release; Enhanced in vivo wound healing process in infectious wound models.	[132]
Doxorubicin	Carrageenan	Average particle diameter, 1–5 μm; Porous structures with pore sizes of 30 nm; 13% cell viability of the human osteosarcoma MG-63 cell line after microparticle administration.	[133]
Lappaconitine	Carrageenan	pH-sensitive microparticle with a drug loading rate of 26% and faster drug release in an acidic environment.	[134]
Rosmarinic acid	Carrageenan	Cationic microparticles with +23 mV zeta potential values; Effective against gram-negative bacteria and some fungi species; Sustained drug release.	[135]
Ciprofloxacin	Carrageenan, Chondroitin	Almost spherical particles with rough surfaces and sizes below 25 μm; Suitable for ocular administration; Good mucoadhesive properties.	[136]
Epidermal growth factor	Ulvan, Chitosan	Porous microstructure with pore sizes of 53 ± 16 μm; Non-toxic behavior; Excellent cell proliferation; Sustained drug release.	[141]
Gentamicin sulphate	Hyaluronic acid	Mean particle size of 9.91 ± 1.57 μm; Drug loading rate of 46.90 ± 0.53%; Good mucoadhesive properties.	[80]
Gentamicin sulphate	Hyaluronic acid, Chitosan	Chitosan microparticles coated with hyaluronic acid/chitosan multilayers; Sustained in vitro drug release due to the barrier effect of the coating.	[149]
Vancomycin	Hyaluronic acid	Microparticles with spherical shapes and porous structures; Controllable and sustained drug release profiles up to 168 h.	[151]
Budesonide	Hyaluronic acid	Particle size, 6.3 μm; Drug loading, 21%; Encapsulation efficiency, 91.5%; Prolonged retention on the surface of the porcine tracheal tube, owing to good mucoadhesion.	[152]
Salbutamol sulfate	Hyaluronic acid	Enhanced biomucoadhesive property in vitro; longer drug pulmonary retention and reduced systemic exposure in vivo.	[153]

## 3. Challenges in the Preparation of Polysaccharide Microparticles

**Figure 3:**
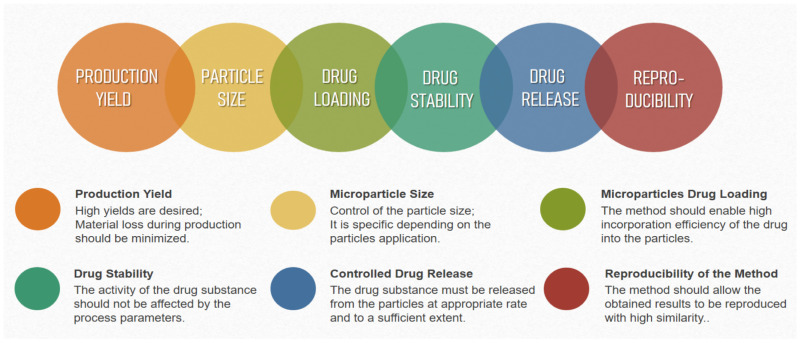
Requirements for microparticle production methods. Created by biorender.com (publication license accessed on 22 June 2023).

There are different techniques for obtaining microparticles from marine polysaccharides. The choice of a preparation method for developing drug-delivery systems depends on various factors such as nature of the polymer carrier, properties of the drug substances included in the particles, route of administration of the dosage form, and so on. The production technique used, in turn, has an impact on many of the characteristics of the particles obtained, as follows: average size, particle size distribution, surface morphology, possible interactions and modifications of the substances during processing, and so on. (Figure 3). Therefore, the technological parameters of the preparation method, which can be varied, should be well known, as should the possibilities for their optimization to successfully prepare microparticles with the desired characteristics.

### 3.1. Emulsification Techniques

These are some of the most commonly used techniques for obtaining drug-loaded polysaccharide microparticles. The polymer and the drug substance are usually dissolved in an organic solvent and the resultant solution is emulsified in an aqueous medium. Microparticles are formed via the subsequent evaporation of the dispersed phase solvent [154]. The classical emulsification method involving the formation of the O/W emulsion is unsuitable for incorporating hydrophilic drugs because they may not dissolve well in the organic phase. The drugs can diffuse into the dispersed medium during emulsification, resulting in high drug loss and limited drug loading efficiency. To overcome this issue, different modifications of the method have been developed. Many authors have suggested using double or multiple emulsions (W/O/W and less often O/W/O). Regarding their preparation, emulsification can be carried out in one or two stages. The first stage involves heating an emulsion composed of a non-ionic emulsifier or a mixture of different emulsifiers, resulting in phase inversion and the formation of a multiple emulsion [155]. However, double emulsions are more often obtained through a two-stage emulsification process. In the first step, an internal (W/O) emulsion is formed using a lipophilic emulsifier under intense homogenization. In the next step, the primary W/O emulsion is dispersed during the aqueous (outer) phase using a hydrophilic emulsifier and slow stirring [156].

The factors influencing the characteristics of the polysaccharide microparticles obtained via the emulsion technique are related to the composition of the prepared emulsion (polymer concentration, type and quantity of the emulsifier), as well as to some technological parameters of the process (stirring rate, stirring duration, temperature, etc.). A higher polysaccharide concentration increases the viscosity of the dispersed phase in which it is used. This may result in the formation of larger droplets and bigger microparticles, respectively [157]. Increasing the viscosity of the inner or outer aqueous phases in multiple emulsions can limit the water movement between the two media and prevent droplet fusion. Moreover, gelling the internal phase may improve the emulsification efficiency and enhance drug loading in the microparticles. Alginates, natural gums, or pectin are commonly used as viscosity-enhancing agents [158].

In the emulsification process, surfactants perform two main functions The first one is to reduce the interfacial tension between the aqueous and oil phases, facilitating the dispersion of the viscous solution of the polymer. The second task involves stabilizing emulsion droplets and limiting their coalescence [159]. Various surfactants can be used for emulsifying polysaccharide solutions. The most widely used emulsifiers for stabilizing microemulsions are non-ionic sorbitan esters—Tween 20, Tween 80, Span 80, Span 85, and their combinations. Their concentrations are usually in the range of 1–3% [2]. To stabilize O/W emulsions, emulsifiers with hydrophilic–lipophilic balance (HLB) values between 6 and 16 are used, whereas for W/O emulsions the HLB values are between 2 and 7. A combination of hydrophilic and lipophilic emulsifiers is commonly used to achieve an optimal HLB. Since the emulsifier is adsorbed on the surface of the droplets of the dispersed phase, and as it forms a film to prevent their fusion, very low concentrations of it can lead to the incomplete coating of the droplets, resulting in physical instability. The coalescence of the microdroplets may be responsible for the formation of larger microparticles. On the other hand, high surfactant concentrations are also not recommended, because they may hinder polymer cross-linking, and therefore, the conversion of drops into particles [160,161]. 

In addition to factors related to the composition of the reaction system, the technological parameters of the emulsification process also affect the characteristics of the resulting microparticles. Studies have shown that increasing the medium’s stirring rate can reduce the resulting microstructures’ size. More energy is included in the system, providing a more efficient dispersion of the phase in the medium and forming finer droplets [162]. Authors have found that a high stirring rate can produce irregularly shaped structures due to particle aggregation [163,164]. Therefore, to obtain satisfactory results, it is necessary to choose a stirring rate tailored to the particular composition, and the desired application, of the microparticles. When preparing multiple emulsions via two-stage emulsification, high-speed homogenization is applied only when the primary emulsion is obtained. Prolonged emulsification at high stirring rates can lead to the incorporation of air into the system. During the second stage, stirring should be slow, because otherwise a simple emulsion will be obtained instead of multiple ones [165].

The preparation of polysaccharide microparticles via the emulsion technique, with solvent evaporation, is a significantly longer process compared with other microencapsulation methods. Its duration depends on the evaporation rate of the solvent used. System heating is usually required, which can endanger the drug’s stability. Moreover, prolonged drying is necessary to completely remove the organic solvent from the resulting microparticles [166]. Sometimes high temperatures cannot be applied due to the polymer’s glass transition. In some cases, the drying process may take more than a week.

Various methods for formulating microemulsions have been studied, which aim to optimize the emulsification process, such as ultrasound emulsification, high-pressure homogenization, and so on. [167]. Despite their high efficiency, these techniques cannot allow for the precise control of the average size of the resulting droplets, and therefore, the formulated final polymer microparticles. In addition, the applied high sheer forces may negatively affect the activity of the drug substances used [168]. In recent decades, new emulsification techniques (membrane emulsification, microchannel emulsification, microfluidic approaches) have been proposed to formulate emulsions, with uniform droplet sizes, by applying low sheer forces to disperse the internal phase into the continuous external medium using microchannels or membrane pores [169]. These preparation strategies are suitable for obtaining droplets of uniform sizes, shapes, and internal structures, thus reducing the use of surfactants and allowing uniform drug distribution, high drug incorporation efficiency, and better reproducibility [155].

In microchannel systems, the size of the resulting droplets, called microparticles, is mainly determined by the flow rate of the two phases. In general, polymer microstructures of smaller dimensions are obtained when accelerating the flow of the dispersed medium and reducing the speed of the dispersed phase feed. The diameter of the particles is also affected by the interfacial tension—the smaller it is, the smaller the resulting droplets. The viscosity of the two immiscible liquid phases is another important parameter. If the viscosity of the dispersed medium is higher and the viscosity of the dispersed phase is lower, the resultant microparticles are usually smaller in size. Smaller particles are also produced by reducing the dimensions of the microchannel systems. The droplets generated through microchannel systems typically range in diameter, from a few micrometers to 100 μm [170,171].

Microporous membrane systems offer the possibility of obtaining emulsions with a narrow droplet size distribution, without the need to apply high mechanical pressure and bring significant energy into the system, compared with conventional mechanical emulsification techniques. The dispersed phase is pushed through the pores of a membrane into the continuous medium at an applied pressure of about 0.5–5 bar of nitrogen, with droplets generated on the membrane surface [172]. The diameter of the membrane pores significantly influences the size of the resulting microstructures [173]. The droplet size can vary from 2 to 10 times the pore diameter, depending on the interfacial tension between the flux from the dispersed phase, the membrane surface, and the dispersed medium. The proposed membranes, with a pore size of 0.49 to 40 μm, allow for microparticles to be obtained, ranging in size from 10 μm to 100 μm [174]. An alternative to this method involves a using so-called premix-membrane, which can increase the production rate, and microspheres with a 1–2 μm size can be obtained [175]. A classical O/W emulsion is prepared via stirring, then, it is repeatedly conducted through a membrane to obtain microstructures with sufficiently narrow size distributions. A disadvantage of the microporous membrane emulsification is the possibility of the fusion of droplets, which mainly depends on the porosity of the membrane used. The porosity also determines the distance between two adjacent pores. This distance should be enough to ensure that neighboring droplets do not get too close to each other, leading to coalescence [176]. To limit the fusion of the microdroplets, cross-flow membrane emulsification can be used. A continuous phase is fed into the countercurrent, to the dispersed medium, to ensure a more efficient collection of droplets formed on the membrane openings. The microstructures break off before becoming large enough to separate on their own, spontaneously, and therefore, they remain smaller compared with the membrane’s pore diameter [177]. For rapid droplet separation, ultrasonic or vibrational membrane emulsification can be used, in which the membrane performs vibrations of a certain frequency [178].

### 3.2. Spraying Techniques

Spray drying is a widely applied method in which a powder formulation is obtained from aqueous or organic solutions, dispersions, emulsions, or suspensions, as a final product. It is a fast and reproducible microparticle production technology that allows for easy planning. Spray drying is a single-step method for producing powders, transforming the starting liquid material directly into dry particles. The process can be divided into three stages, as follows: pumping the starting liquid material through a nozzle and producing fine droplets; droplets come into contact with a gas stream at a high temperature, causing the liquid to evaporate; and the separation and collection of dry particles as a final product [179,180]. The sample is sprayed in the form of small sized droplets. Therefore, the liquid phase’s surface area/volume ratio is significantly increased. The larger surface area leads to the rapid evaporation of the solvent. The contact time between the formulated droplets and the hot gas required to obtain solid particles is only a few seconds. This time is considered too short to affect the drug substances’ stability. The sample never reaches the inlet gas temperature. During the drying process, the moisture that evaporates keeps the temperature of the droplets lower, as compared with the inlet one. As the droplets are dried, the gas is gradually cooled until equilibrium is reached between its temperature and that of the resultant dry particles [181,182]. The technological process parameters which affect the characteristics of the resultant microparticles are as follows: the concentration of the starting material (polymer and drug substance); the rate at which the peristaltic pump carries the sample to the nozzle of the apparatus; the amount of compressed gas required to disperse the sample into droplets; the temperature of the gas used for the drying process; and the rate of the aspiration [183,184]. 

Despite the many advantages of spray drying, this technique also has some limitations. Traditional spray dryers are designed for the industrial production of microspheres. Therefore, the yields obtained are relatively low in laboratory conditions when working with small samples. Typically, the production yields are in the range of 20–70%. The main reason for the small yield is the adherence of the sprayed droplets onto the walls of the spray dryer chamber, especially when working with adhesive polymers such as polysaccharides. The amount of material lost during drying is relatively constant. Therefore, a higher yield is observed when working with larger sample volumes. Due to the insufficient separation ability of the cyclone, a large number of the finest particles is usually aspired and carried out of the system with the exhaust air. The need for expensive equipment is another disadvantage of the spray drying technique. The nozzle of the apparatus can easily become clogged, especially if solutions of polysaccharides with a high viscosity are sprayed. The adherence of the pulverized material to the walls of the spray dryer chamber is associated not only with production loss and low yields, but also with cleaning expenses [185,186].

Spray freeze-drying is a relatively newer drying technique which consists of several steps—droplet formation, freezing, and sublimation. The method allows polysaccharide microparticles with good physico-mechanical characteristics and stability to be obtained [187]. It combines two drying techniques, namely, spray drying and freeze-drying (lyophilisation). Spray freeze-drying involves breaking the liquid sample into droplets, solidifying the droplets via direct contact with a freezing agent (cryogen), and sublimating the solidified droplets at a very low temperature and pressure [188]. Different nozzles can be used to disperse the sample—nozzles for two, three, or four liquids. Ultrasonic nozzles allow for the more precise control over the size of the resulting microparticles, and those four liquids are suitable for encapsulating drug substances with limited water solubility [189]. The spraying stage determines the size of the microparticles, and the main factors influencing the process are as follows: the viscosity of the sprayed material, the spraying rate, and energy used. The next step involves freezing the droplets at low temperatures with a cryogenic agent such as liquid nitrogen. The method is suitable for encapsulating thermosensitive active substances, unlike spray drying, in which a high temperature evaporates the solvent. On the other hand, compared with freeze-drying, the spray freeze-drying technological process is significantly shorter and the resultant microparticles have the spherical shape of the initial droplets. In most cases, the final microparticles are highly porous, allowing rapid rehydration [190].

Jet break-up methods are another technique that can be used to formulate polysaccharide microparticles. Their mechanism involves feeding a polymer solution through a nozzle at a constant rate, forming a laminar jet, breaking the jet, and obtaining a chain of microdroplets with very close dimensions. The droplets fall into a medium with a crosslinking (gelling) agent, in which the polymer becomes insoluble and the droplets form solid microparticles [171]. Jet break-up can be accomplished via electrospraying, jet-cutting, and jet vibration excitation.

Regarding the electrospraying method, an electric field is created between the nozzle and the solution, in which the polymer droplets solidify. Under the impact of electrostatic forces, the liquid breaks up into fine droplets with narrow distributions in size. There are two methods, as follows: the dropping and jetting techniques. During dropping, the polymer solution is slowly fed through the nozzle, wherein a low voltage of up to 4 kV is applied, breaking the liquid into microdroplets. In this sense, microspheres between 500 and 1500 μm are obtained. Smaller microparticles can be designed under increased voltages and using a smaller nozzle, however, the resulting particles may then have a broader size distribution [191,192]. Regarding the jetting technique, the polymer solution is fed at a greater rate, in which it is not dropped directly, but a continuous jet is formed. A high electrical voltage is required to break the jet into small droplets. With this approach, monodisperse microparticles with dimensions of 1–15 μm can be obtained [193,194]. An advantage of electrospraying concerns the use of affordable and easy-to-use equipment. Furthermore, regarding the jet technique, the size of the microspheres can be controlled via the electric current force, regardless of the nozzle diameter. A larger nozzle can be used, avoiding the problem of clogging, without affecting the particle diameter [195]. Moreover, the electric charge of the droplets prevents their fusion, and surfactants are not required. The method is also suitable when working with proteins and cells, which are stable under electrical forces up to 10 kV [192,195].

Instead of spraying, the feed jet can be cut into equal segments using a cutting tool consisting of several metal wires (jet-cutting technique). The method has been applied to obtain chitosan and alginate microparticles [196]. The diameter of the resulting droplets is determined by the number of cutting wires, the number of rotations of the cutting tool, and the flow rate of the polysaccharide solution through the nozzle. This technique allows the production of microparticles even from viscous liquids with a viscosity above 500 mPa.s, at a high feed rate (10–30 m/s). The size of the resultant particles is usually above 200 μm. Another jet-cutting technique involves the use of a rotating disc [197]. The polymer solution is fed onto the disc, and under the centrifugal force, it is oriented towards the periphery, forming microstructures. The obtained microparticles, are collected in a flat receiver, divided into sectors, in accordance with their size. Polysaccharide microparticles of uniform dimensions can be produced by controlling the process parameters.

Regarding the jet vibration excitation method, the jet is broken into homogeneous segments using generating vibrations. The horizontal oscillations applied to the polymer solution cause surface instability, breaking the liquid flow into uniform droplets. The main process parameters that affect the characteristics of the resulting polysaccharide microparticles are as follows: frequency and wavelength generated, jet diameter, viscosity, and interfacial tension of the polymer solution. Nozzles with smaller diameters and higher frequencies can increase the possibility of the fusion of generated drops. The wave frequency is generally kept as low as possible to avoid the formation of satellite droplets, leading to broader size distribution. The longer the length of the generated wave, the less likely the fusion of the drops. By increasing the concentration of the polysaccharide solution and the jet feed rate, an increase in droplet size can be achieved [198].

### 3.3. Coacervation Techniques

Phase separation (coacervation) is one of the oldest techniques for obtaining polymer microparticles. Coacervation is classified as a physico-chemical method of microencapsulation. Polymer microparticles are formulated by mixing two different colloidal phases, one rich in polymeric structures (coacervation phase) and the other polymer-free (equilibrium phase). Both hydrophilic and hydrophobic drug substances, in the form of liquid emulsions or suspensions, may be microencapsulated, and they must be insoluble or slightly soluble in the solution of the polymer that constructs the microparticles’ matrix/coatings [199]. Coacervation can be simple or complex depending on the mechanism by which phase separation occurs.

Simple coacervation involves using only one type of polymer, the solubility of which is lowered by changing the pH, or adding an electrolyte or a second solvent to the system in which the polymer is insoluble. As a result, phase separation and particle formation occur. For example, chitosan microspheres can be obtained through simple coacervation by adding a solution of chitosan in acetic acid dropwise in a solution of NaOH under continuous stirring conditions. The change in pH leads to the precipitation of the polymer and the formation of microspheres that can be further stabilized by the addition of a crosslinking agent [200].

Complex coacervation is another effective method for microencapsulating drug substances in polysaccharide carriers. In this technique, microspheres are formed by ionic interactions between solutions of oppositely charged polymers. Phase separation occurs in an aqueous medium due to the attraction between the opposite charges of the polymer molecules and the formation of polyelectrolyte complexes [5]. An example of complex coacervation concerns the binding between the negatively charged carboxylic groups of alginate with the positively charged amino acids from the chitosan structure [201,202]. Due to the ionic interaction between alginate and chitosan, the solubility of the polymers decreases. Alginates dissolve in alkaline media and they are insoluble in acids, whereas it is the opposite for chitosan. Two approaches can be used to obtain microparticles from the chitosan–alginate complex, as follows: mixing the solutions of both polymers, adding one to the other via stirring, or coating the resultant particles from one of the polymers with particles obtained from the other one [203].

Studies have reported that by increasing the polymer concentration and increasing the viscosity of the polymer solution, the microparticles obtained via the phase separation method have a larger size and higher drug entrapment efficiency, respectively [204]. Low polymer concentrations can lead to the formation of microstructures with low densities, broad size distributions, and the rapid release of encapsulated drug substances [205]. A key point in complex coacervation is to provide a pH in which the two polymers are oppositely charged. The optimal pH to achieve effective phase separation depends on the nature of the polymers used [199]. For example, regarding interactions between chitosan and alginate, to formulate a polymer matrix, a medium with a pH of about five is needed. Under alkaline conditions, chitosan is not positively charged and cannot bind to the anionic polymer. At an acidic pH, the carboxylic groups in the alginate structure are protonated, forming an insoluble layer of alginic acid on the surface of the microparticles. This prevents liquid from entering the particles from the external environment, thus limiting their swelling. On the other hand, at an acidic pH, the amino groups in the chitosan structure are converted into soluble NH_4_^+^ groups that can interact with the protonated carboxylic groups of the alginate [203].

The rate and duration of stirring in the coacervation process also influences the size of the polysaccharide microparticles and their size distribution. Studies have shown that if a high stirring rate is applied to the system, smaller microspheres are obtained, and the efficiency of drug incorporation in the polymer matrix may be lowered. As the stirring time increases, the microparticle size rises too, but the drug incorporation efficiency may decrease due to the increased crosslinking time of the polymer. On the other hand, insufficient crosslinking time can also lead to low encapsulation efficiency. Therefore, the reaction time should be optimized, depending on the polysaccharides and drug substances used, to ensure the preparation of microparticles of the desired size and high drug incorporation efficiency [204].

## 4. Biomedical Application Perspectives of Polysaccharide Microparticles

**Figure 4:**
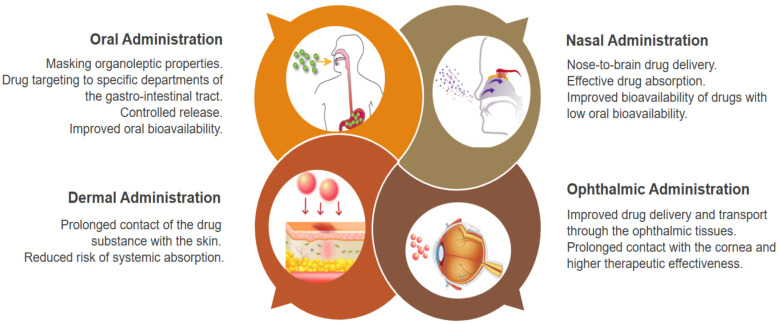
Administration routes for polysaccharide microparticles as drug delivery systems. Created by biorender.com (publication license accessed on 22 June 2023).

As mentioned above, polysaccharide microparticles are suitable drug-delivery systems, with modified drug release systems intended for oral, nasal, dermal, and ophthalmic administration (Figure 4).

By designing polysaccharide microstructures as drug-delivery systems, some of the limitations concerning oral routes of drug administration can be overcome, such as the unpleasant taste of the drug substance, the irritating effect on the gastrointestinal mucosa, drug degradation via gastric digestive enzymes, instability in the acidic pH of the stomach, incomplete absorption, and impacted metabolism due to the first-pass effect. Such systems can provide an increased stability of the drug substance, masking its organoleptic properties, drug targeting to certain sites of the gastrointestinal tract, controlled drug release, and increased oral bioavailability [206]. The possible pathways for microparticles to pass through the intestinal barrier have been extensively studied [207]. There are two main mechanisms for transport, as follows: paracellular passage through the intercellular space of adjacent cells, and transcellular passage through the cell membranes of the intestinal mucosa. Studies on cell cultures have shown that using polysaccharides, such as chitosan and alginate, can improve the intercellular passage of microparticles in the small intestines [208]. The transcellular mechanism is the main route by which microparticles can pass through the intestinal barrier. Microfold (M) cells from the follicle-associated epithelium (FAE), covering Peyer’s patch, have been outlined as essential for microparticle transport. M-cells are of a phagocytic type, they are specialized in the ingestion of particles, including microorganisms, antigens, and so on. Microparticles adhere to M cells, which absorb them via endocytosis; then, they are transported to the basolateral regions, where they are released via exocytosis [209]. Particle properties, which may affect transcellular transport and M-cell binding are as follows: particle size, charge, hydrophilic/hydrophobic characteristics, and presence of specific ligands on the particle surface [210]. Studies have shown that only microparticles that are smaller than 10 μm can pass through Peyer’s patches, whereas transport through intercellular spaces is also possible for structures of larger sizes (up to 150 μm) [209].

In addition to increasing oral drug bioavailability, microencapsulation is a widely used approach to ensure the stability of biologically active substances such as vitamins, omega acids, essential oils, probiotic bacteria, prebiotics, enzymes, polyphenols, carotenoids, and so on. The use of such active substances is often limited due to their sensitivity to temperature, pH, light, oxygen, and degradation via enzymes. Therefore, they must be protected from environmental factors to minimize or prevent their degradation. Microencapsulation is a convenient and effective method to maintain and improve the biological and functional characteristics of active substances sensitive to processes such as oxidation, photodegradation, or evaporation, and achieve their controlled release. Many studies are focused on polysaccharides, such as those focused on controlled-release vehicles for various vitamins (ascorbic acid [211], retinol [212], cobalamin [213], and thiamin) [214]. Alginate, chitosan, and carrageenan have successfully been used to microencapsulate various probiotic strains [215,216,217] using the applied production techniques which preserve the vitality of the microorganisms. Microencapsulation is also a key approach which increases the stability of essential oils. Polysaccharide carriers can reduce the evaporation of essential oils, mask its strong taste, and control its release. Successful microencapsulation should result in particles that can retain the essential oil in their cores without allowing migration to the surface [218].

Polysaccharide microparticles can perform an essential role as protein and peptide delivery systems. The rapid development of modern biotechnology and techniques for the preparation of recombinant DNA have stimulated the development of more protein and peptide drugs worldwide. However, these therapeutic agents are usually characterized by a short biological half-life, as they are easily hydrolyzed or degraded by enzymes in vivo. In addition, their lipophilicity and high molecular weight may hinder their oral absorption and bioavailability. Via microencapsulation, proteins/peptides can be coated in a polymer capsule or incorporated into a matrix, which can protect them from enzymatic degradation as well as enhance their absorption in the small intestines, achieving high drug concentrations over a prolonged period of time. Using appropriate polysaccharides and encapsulation methods, microcarriers for the oral delivery of biodrugs can be designed, which is comparable to parenteral formulations [219]. Despite the many studies related to developing and applying microencapsulated protein structures, few products have been released on the pharmaceutical market [220]. The main challenges, in this regard, are related to achieving size uniformity of the microparticles and preserving the activity of the therapeutic agents during the production of the formulations. It is not easy to control the microspheres’/microcapsules’ size and size distribution, which makes it difficult to ensure reproducibility on an industrial production scale. There is also a risk of inactivation, with regard to the proteins/peptides during the microencapsulation process, and afterwards, during their storage [221]. Therefore, choosing an optimal production technology and a suitable carrier are essential for designing polymer systems for protein and peptide delivery. Such systems, with increased oral drug bioavailability and improved therapeutic activities, for example, have been developed using alginate and chitosan carriers for the oral delivery of insulin [222,223], peptides with antihypertensive effects [224], and other biologically active peptides. 

The nasal route of administration can be used as an alternative to the oral and parenteral administration of vaccines, peptides, hormones, and other drug substances with systemic action properties. Moreover, this drug administration route is promising for direct drug transport to the central nervous system (CNS), and it achieves nose-to-brain delivery. Intranasally administered substances can reach the CNS within a few minutes, due to the unique connection between the nasal cavity and the brain, via the olfactory nerves [225]. Microspheres based on polysaccharides with marine origins, such as chitosan and alginate, have been thoroughly studied as carriers for nasal applications. Combining the advantages of the microcarriers, with the mucoadhesive properties of these polymers, brings additional benefits such as more effective absorption and increased bioavailability due to the large total surface area of the particles, better contact with the nasal mucosa, and specific targeting to the absorption site. The development of nasal polymer microparticles is a successful strategy for the delivery of drugs with low oral bioavailability (e.g., carvedilol [105] and verapamil [85]; antimigraine therapeutic agents such as zolmitriptan, sumatriptan, and dihydroergotamine [226,227]; as well as some high molecular weight peptides such as insulin [87,228]).

In recent years, drug-delivery systems based on a microparticle design have been reported as a successful alternative to conventional therapeutic approaches in ophthalmology. These systems offer an opportunity to improve drug delivery and transport via the ophthalmic tissues [70]. Due to their small size, nanostructures are rapidly removed through the conjunctival, scleral, and other periocular circulation systems, and they cannot ensure a constant drug concentration [229]. The poor bioavailability of topically administered drugs limits their access to intraocular tissues. Systemic administration requires high doses to achieve adequate therapeutic levels of the drug in the eye, with the risk of systemic adverse effects. Repeated applications are usually required for successful therapy, causing much inconvenience. Microparticles can release the active substance for longer periods compared with nanoparticles. Microparticles are preferred, especially in chronic diseases that require low concentrations of the active substances for an extended period of time. Nanoparticles are associated with a higher degree of penetration, and they can help improve drug bioavailability, but they can cause high concentrations of the administered therapeutic agent in the retina, which can be toxic [229,230,231].

Microparticles can be applied topically as a suspension, or they can be injected using a needle (30–32 G), in minimally invasive procedures [230]. Furthermore, most natural polysaccharide microcarriers are characterized by a high degree of safety, biocompatibility, and lack of immunogenicity. The use of mucoadhesive polymers to prolong the contact of particles with the cornea is among one of the main strategies for achieving higher therapeutic efficacy after topical application [231]. Microcarriers can improve drug bioavailability in the eyes, as well as minimize systemic absorption. The microparticle size intended for the anterior segment of the eye (cornea, iris, lens, ciliary body) should be smaller than 25 μm [136]. It has been shown that particles smaller than 10 μm do not cause mechanical irritation and injury to the eyes. At the same time, structures of larger sizes can scratch the highly innervated eye surface during blinking, and they can cause discomfort to the patient [232]. Polymer microparticles can also be promising drug-delivery systems when treating pathologies affecting the posterior segment of the eye (sclera, choroid, retina, vitreous). Polymer microsystems for intravitreal drug delivery can limit the risk of drug side effects, as they can release the active substances over a long period of time. They must be designed to ensure the controlled release of the drug substance that is incorporated into them. Biodegradable polymeric microparticles can be administered intravitreally in the form of a suspension using conventional needles. To be administered via injection into the vitreous body, they must have dimensions smaller than 75 μm. [233]. The applied microparticles usually exhibit a tendency to agglomerate, which can lead to the formation of zones with increased densities, in which, the particles are deposited. This creates a “pseudo-implant” with specific physicochemical and biopharmaceutical properties, and a longer retention time [70]. Regarding ophthalmic formulations, the drug-delivery systems for administration in the eyes must necessarily meet the sterility requirement. The sterilization process must not jeopardize the stability of the microparticles, nor alter their physicochemical properties and therapeutic efficacy. However, the sterility requirement is often neglected in the studies reported in the literature [231].

The use of microparticles as drug-delivery systems is a rational approach to achieve a local effect after dermal application, when the drug substance should act on the surface of the epidermis. The mechanism of drug release from the microparticles ensures prolonged contact of the drug substance with the skin after the dermal application of the formulation, reducing the risk of systemic absorption. Due to their microscopic size, microparticles do not pass through the epidermis, but are localized in the skin pores, where they can release the drug substances in a controlled manner. Polymer microparticles have been used as carriers of anti-inflammatory agents and growth factors for skin injuries and other dermal conditions [68].

Novel and improved microcarriers for drug delivery are constantly being proposed and outlined as successful pharmaceutical formulations. However, most reported studies are only based on in vitro tests, and they lack thorough in vivo analyses. Further research and clinical trials are needed to evaluate the efficiency and safety of polysaccharide microparticles as therapeutic systems, and to enable their utilization on the pharmaceutical market as final pharmaceutical products. Although most marine polysaccharides are permitted for biomedical applications, there are still major limitations regarding their use as pharmaceuticals in clinical trials. Some primary regulatory issues are attributed to their source and characterization, especially for animal-derived materials, such as chitosan, chondroitin, and hyaluronic acid. Purity is another critical parameter for marine polysaccharides as they may have impurities such as high bioburden, bacterial, and protein contamination. Hypersensitivity to seafood is a common allergy. Therefore, total protein content estimation is a significant concern. Manufacturing guidelines for pharmaceutical-grade marine polysaccharides, with mandatory tests for hypersensitivity, endotoxin levels, immunogenicity, systemic toxicity, and purity, should be established to guarantee the requirements for a pharmaceutical product’s polymer quality and safety [234].

## 5. Conclusions

Currently, micro-technologies have been intensively investigated worldwide. The inherent natural properties of marine polysaccharides, such as biodegradability and biocompatibility, make marine polysaccharide-based microcarriers a high potential platform for developing drug delivery systems. Although drug delivery via polysaccharide microparticles has been the focus of much research in recent years, this area of pharmaceutical technology is still undergoing rapid development, and the marine polysaccharide-based drug formulations still have a long way to go before clinical applications can occur. The progress achieved should be further expanded upon with more in-depth studies that will allow for the maximum utilization of such beneficial systems, revealing new opportunities for improved drug delivery.

## Figures and Tables

**Table 2:** Alginate-based microparticles as drug delivery systems.

Active Substance	Administration	Reported Results	Reference
Ranitidine	Oral	Spray-dried microspheres with a smooth surface, narrow particle size distribution; Drug loading of 70.9%; Prolonged drug release in accordance with first-order kinetics.	[93]
Acyclovir	Oral	An average particle size of 70.60 µm; Drug entrapment efficiency of 51−80%; Good mucoadhesion (66%); Prolonged drug release in accordance with Peppa’s kinetic model.	[94]
Isoniazid	Oral	An average particle size of 3.719 μm; Drug entrapment efficiency, 40–91%; Prolonged retention in the small intestine—up to 24 h post oral administration.	[95]
Isoniazid	Oral	Spherical microspheres; Drug encapsulation efficiency, 93%; High bioadhesion, 81%; Improved drug oral bioavailability (increased C_max_, T_max_, t_1/2_, and AUC).	[96]
Metformin hydrochloride	Oral	Enhanced drug hypoglycemic activity evaluated in vitro, based on glucose uptake in *Saccharomyces cerevisiae* cells and α-amylase inhibition tests.	[97]
Caffeine	Oral	Spray-dried, cross-linked microparticles with sizes in the range of 4–7 μm; Increased stability with regard to digestion, and decreased amounts of drug released within the simulated gastric fluid.	[98]
Insulin	Oral	Mean particle diameter, 2.1 ± 0.3 μm; Protein encapsulation efficiency, 38%; 88% of the released insulin from the particles was bioactive.	[99]
Diclofenac sodium	Oral	Production yield, 80–97%; Drug entrapment efficiency, 66–96%; Sustained in vitro drug release following zero order kinetics.	[100]
Indomethacin	Oral	Controlled drug release; Increased drug t_1/2_ and AUC values evaluated using HPLC technique in vivo on rabbits.	[101]
Aceclofenac	Oral	A drug entrapment efficiency of 86–97%; Prolonged drug release in accordance with Power law kinetics and case-II (or) anomalous transport mechanisms.	[102]
Piroxicam	Oral	Spherical, free-flowing microspheres with average particle sizes of 950 μm; Sustained drug release within 22 h via the Non-Fickian diffusion mechanism.	[103]
Metoprolol tartrate	Nasal	Matrix-diffusion controlled drug delivery; Improved drug therapeutic efficacy—sustained and controlled inhibition of isoprenaline-induced tachycardia in vivo.	[104]
Carvedilol	Nasal	Mean particle size of 26–54 µm; Encapsulation efficiency, 36–56%; Mucoadhesion on sheep nasal mucosa, 69–85%; Non-Fickian or anomalous type of transport release.	[105]

## Data Availability

Not applicable.
